# Hydroxybenzoic Acids Are Significant Contributors to the Antioxidant Effect of Borututu Bark, *Cochlospermum angolensis* Welw. ex Oliv.

**DOI:** 10.3390/antiox6010009

**Published:** 2017-01-28

**Authors:** Ehab A. Abourashed, Hao Wen Fu

**Affiliations:** College of Pharmacy, Chicago State University, Chicago, IL 60628, USA; hfu@csu.edu

**Keywords:** flash chromatography, centrifugal thin-layer chromatography, phenolic acids, apocarotenoids, antioxidants, DPPH scavenging, HPLC fingerprinting

## Abstract

Borututu (*Cochlospermum angolensis*) is an African tree whose bark has recently emerged as a herbal dietary supplement with claims for antioxidant activity. In order to substantiate the claimed activity of borututu supplements, we performed an activity-guided fractionation of the total extract utilizing a 1,1-diphenyl-2-picrylhydrazyl (DPPH) free radical scavenging assay. Subsequent flash and centrifugal chromatography resulted in the isolation of gallic acid (**1**) and protocatechuic acid (**2**) as the main antioxidant constituents. Two apocarotenoids and one flavonoid were also isolated from the chloroform fraction and were identified as cochloxanthin (**3**), dihydrocochloxanthin (**4**), and 7,4′-dimethyltaxifolin (**5**), respectively. A High-performance liquid chromatography (HPLC) method was also developed for fingerprinting borututu samples, with Compounds **1**–**4** suggested as chemical markers for quality control purposes.

## 1. Introduction

Borututu (*Cochlospermum angolensis* Welw. ex Oliv., family Bixaceae or Cochlospermaceae) is an African tree indigenous to Angola (West Africa) and The Democratic Republic of Congo (Central Africa). The bark of borututu tree has been used in the preparation of traditional local beverages and as a remedy for various diseases [1,2]. In countries where malaria is most prevalent, borututu root has been studied for the treatment of *Plasmodium falciparum* and *P. berghei* infections [3]. In in vitro experiments conducted by Presber et al. [3], borututu root extract reduced *P. falciparum* parasite multiplication by 50%. Similarly, erythrocytes obtained from mice infected with *P. berghei*, showed immediate inhibition of parasite DNA replication and protein expression after exposure to borututu extract [4]. In a recent study, Ferreres et al. demonstrated a significant antioxidant activity of different borututu extracts, which was attributed to ellagic acid and related analogs [5]. Similarly, Pereira et al. showed that a boiling water infusion of borututu bark showed antioxidant and antitumor activity [2]. However, another study by Costa et al. stated that a borututu tea infusion had the lowest antioxidant activity compared to other herbal teas [6]. In a phytochemical study by Leonardi et al., the volatile oil of *C. angolensis* leaves and roots were reported to contain the sesquiterpenes germacrene D, α-cadinol, 10-epi-cubenol, β-caryophyllene, and isoborneol [7]. Other *Cochlospermum* species, mostly *C. tinctorium*, have also been investigated. For example, extracts of the root and rhizome of *C. tinctorium* have been evaluated for cytotoxic, antimicrobial, antiplasmodial, anti-ulcer, antioxidant, hepatoprotective, and immunomodulating effects [8,9,10,11,12]. Preliminary antibacterial and antifungal evaluation was also performed on the ethanolic leaf extract of Brazilian *C. regium* [13]. Secondary metabolites identified in *C. tinctorium* include triterpenes (alphitolic acid & 3-*O*-*E*-*p*-coumaroylalphitolic acid), apocarotenes (cochloxanthin & dihydrocochloxanthin), as well as triacylbenzenes (e.g., 1,3,5-tri[dodecanyl]- & tri[tetradecanyl]benzenes, also isolated from *C. angolensis*, *C. planchonii*, and *C. tinctorium*) and volatile ketones, such as 1-hydroxytetradecan-3-one [10,14,15,16]. Gas chromatography/mass spectrometry (GC/MS) analysis of essential oil of the leaves, root bark, and root wood of *C. vitifolium* resulted in the identification of α-humulene, β-caryophyllene, β-bisabolene, β-pinene, γ-muurolene, and 1-hydroxy-3-hexadecanone. Additionally, phytochemical analysis of the non-volatile extract revealed excelsin, pinoresinol, narigenin, aromadendrin, gallic acid, triacylbenzene, as well as β-sitosterol and stigmasterol in free form and as d-glucosides [17].

With the recent emergence of borututu bark as a new antioxidant herbal beverage and dietary supplement [1,6,18] and due to the scarcity of phytochemical investigation of this plant, the goal of this study was to utilize high-performance liquid chromatography (HPLC) and an activity-guided approach to determine the most active constituents of borututu bark in a 1,1-diphenyl-2-picrylhydrazyl (DPPH) free-radical scavenging assay. A qualitative HPLC method was developed for (i) fingerprinting borututu crude and commercial products; (ii) determining the major phytochemical markers of the bark; and (iii) verifying the purity of isolated compounds. Gallic acid (**1**) and protocatechuic acid (**2**) were the most active DPPH scavengers identified in the ethyl acetate fraction. The two apocarotenoids cochloxanthin (**3**) and dihydrocochloxanthin (**4**) were also isolated and identified in addition to the flavanoid taxifolin-7,4′-dimethyl ether (**5**). The five isolated compounds are reported in borututu for the first time.

## 2. Materials and Methods

### 2.1. General Procedures

Coarse shredded borututu bark was purchased from Mond Trading Corp. (Toronto, ON, Canada). Other herbal products and dietary supplements were purchased online. DPPH and Trolox were purchased from Sigma (St. Louis, MO, USA). All solvents were of reagent grade (Fisher Scientific, Fair Lawn, NJ, USA). Ultrapure RO water was generated in-house (Barnstead Nanopure, Thermo Scientific, Marietta, OH, USA). HPLC fingerprinting, compound purity, and molecular weight determination were performed on an HPLC system equipped with UV/Vis and single-quadrupole mass detectors (LCMS-2020, Shimadzu, Kyoto, Japan). NMR experiments were run on an ECS-400 spectrometer (JEOL, Tokyo, Japan) using CDCl_3_ and acetone-*d_6_* as solvents.

### 2.2. Preparation of Total Extract and Solvent Fractions

Borututu bark was ground to a fine powder and 500 g was soaked overnight in methanol (3 L × 3). The three batches were filtered, combined, and concentrated under vacuum at 45 °C in a rotary evaporator (model R-215, Buchi, Flawil, Switzerland) to yield 102.8 g of a dark reddish brown residue of total methanolic extract (TME). Four aliquots of TME (57.2 g total, ca. 14.3 g per run) were separately triturated to homogeneity with dry silica, placed in a 100 mL stainless steel cylinder and fractionated using solvents of increasing polarity, viz. *n*-hexane, CHCl_3_, EtOAc, and MeOH, in an accelerated solvent extractor (Dionex ASE 150, Thermo Fisher Scientific, Waltham, MA, USA) with the following settings: temperature, 55 °C; static time, 15 min; rinse volume, 60%; purge time, 100 s; static extraction cycles, 3. Fractions extracted with each solvent were combined and concentrated in a rotavapor at 45 °C then transferred to pre-weighed vials labeled HF, CF, EF, and MF for *n*-hexane, CHCl_3_, EtOAc and MeOH fractions, respectively. Total extracts of reference herbals and spices were prepared by ultrasonicating finely powdered 5 g samples of green tea (GTE), milk thistle (SME), horse apple (BPE) and nutmeg (MFE) in methanol for 15 min followed by filtration and drying at 45 °C under vacuum.

### 2.3. HPLC Fingerprinting

An LC-MS 2020 system (Shimadzu) equipped with a binary pump, auto-injector, UV-Vis detector, and single-quadrupole mass analyzer was utilized to develop a qualitative analytical method for extracts, fractions and pure compounds. Column: HyPurity C_18_, 150 × 4.6 mm, 3 μ (Thermo Scientific, Asheville, NC, USA); guard column: SecurityGuard C_18_, 5 μ (Phenomenex, Torrance, CA, USA); solvent A: 0.1% aqueous formic acid; solvent B: acetonitrile; gradient: 5% B for 1 min, to 100% B in 25 min, 100% B for 1 min, re-equilibrate for 3 min; detection: 254 nm; injection volume: 10 μL; MS: full-scan; negative ion; default interface settings and ion optics; low-resolution molecular weights of isolated compounds determined in extracted-ion mode. All samples were dissolved in methanol and filtered through 45 μ cellulose membranes into HPLC vials before injection.

### 2.4. Chromatographic Isolation and Purification of Compounds

Thin-layer chromatography (TLC) was performed on 60 F_254_ silica-coated aluminum plates (Merck, Darmstadt, Germany), while centrifugal TLC (Chromatotron^®^, 7924T, T-Squared Technology, San Bruno, CA, USA) was performed on silica-coated glass rotors under the following conditions: sorbent silica–CaSO_4_ 3:1 mixture (Sigma), 2 mm thickness (prepared per manufacturer directions). TLC and centrifugal rotor plates were developed with the following solvent mixture: CHCl_3_–MeOH, 9:1 (system A). Chromatotron sample volume: 1 mL of 50 mg/mL solution; eluting solvent: 500 mL of system A; fraction volume: 20 mL (Retriever 500 fraction collector, Teledyne Isco, Lincoln, NE, USA). TLC plates were visualized under visible light and UV (254 and 365 nm), with 0.5% ferric chloride, and/or via baking at 100 °C with 10% methanolic sulfuric acid. Fractions were monitored by TLC, and similar fractions were pooled and concentrated, leading to the isolation of **1** & **2**. For the isolation of **3**–**5**, flash chromatography was performed on Isolera One^®^ (Biotage, Uppsala, Sweden) using 25 g SNAP HP-SIL columns and 3 g samplet cartridges loaded with 200 mg of ethyl acetate fraction (EF) for each run. Solvents: methanol (A) and CHCl_3_ (B); gradient: 0% A for 3 column volumes (3 CVs; 1 CV = 33 mL), 0–20% A (20 CV), 20% A in B (4 CV); flow rate: 25 mL/min; detection at 254 nm; fraction volume: 22 mL; total run time: 40 min. Similar column fractions were pooled and concentrated based on their peak profile which was confirmed by TLC.

### 2.5. DPPH Assay [19]

**Figure 1:**
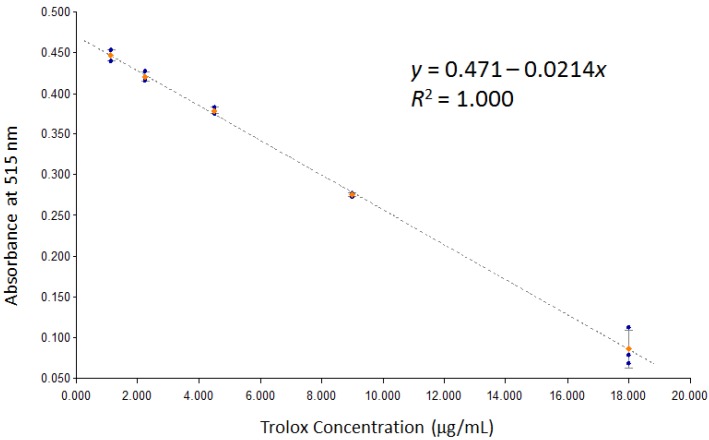
DPPH scavenging calibration curve for Trolox standard.

DPPH stock solution was prepared in a volumetric flask by dissolving 6.25 mg of DPPH in 25 mL of MeOH (25 mg/100 mL, 0.62 mM). Trolox standard solution was prepared by dissolving 4.5 mg of Trolox in 250 mL of methanol (18 μg/mL, 72 μM). A DPPH working solution (0.16 mM) was prepared by a 1:3 dilution of the stock solution. Trolox calibrators were prepared by serial dilution of the stock standard solution to generate 5 concentration levels (1.13–18 μg/mL). Sample stock solutions were prepared at 0.5 mg/mL concentration. Samples were tested at 1:10 dilution and further dilutions were subsequently prepared based on initial runs. Assays were performed in 96-well plates as follows: 100 μL of each blank (MeOH), sample and standard were transferred to specific wells followed by 100 μL of the DPPH working solution (all samples run in triplicates). Each plate was covered and kept in the dark for 30 min, after which it was scanned at 515 nm in an Epoch plate reader (BioTek, Winooski, VT, USA). The concentration of each sample was calculated from the generated calibration curve (Figure 1), and antioxidant activity was recorded as percent Trolox equivalence based on the following formula:

$$\begin{array}{l} \mathrm{Percent Trolox Equivalence of Sample}\;\left(\text{\%}\right)=\\ 100\times \mathrm{Sample Dilution Factor}\times \frac{\mathrm{Trolox Equivalent Concentration}\;(\mathrm{mg}/\mathrm{mL})}{\mathrm{Sample Concentration}\;(\mathrm{mg}/\mathrm{mL})} \end{array}$$

## 3. Results & Discussion

### 3.1. Extraction, Fractionation, and Isolation of Active Compounds

**Figure 2:**
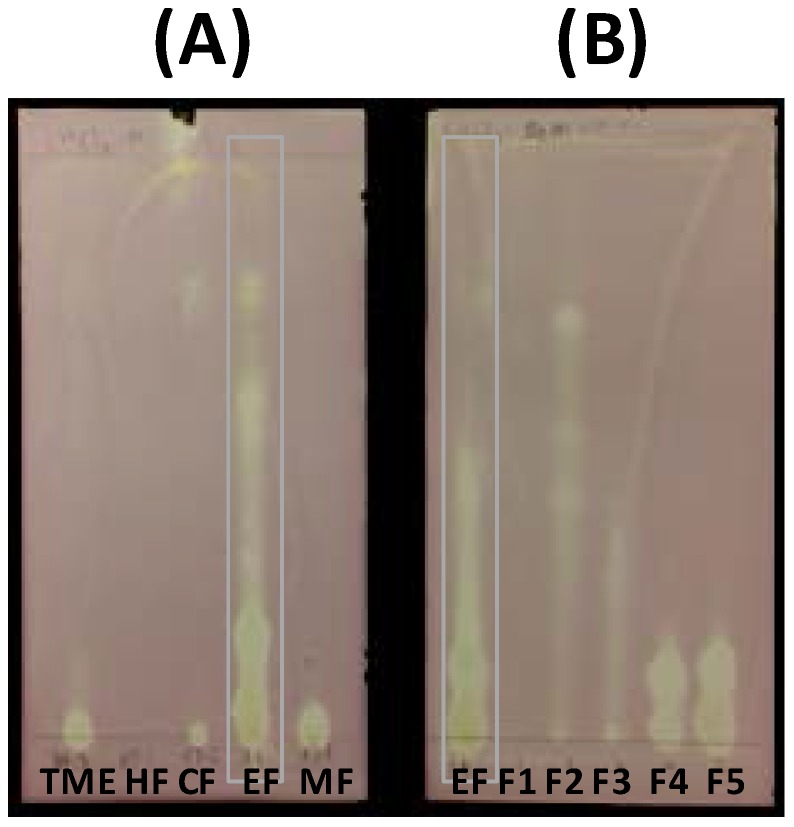
TLC analysis and visualization of active borututu fractions by DPPH dipping. (**A**) Solvent fractions HF, CHCl_3_ fraction (CF), ethyl acetate fraction (EF), and methanol fraction (MF) in comparison to total methanolic extract (TME). (**B**) Flash fractions SF1–5 in comparison to EF.

**Figure 3:**
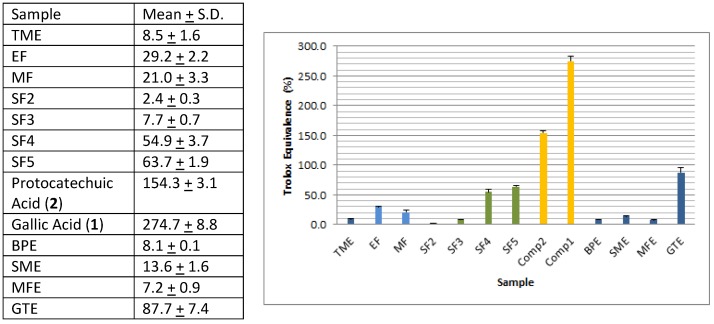
DPPH scavenging activity of total extracts (TME), solvent fractions (EF & MF), column subfractions (SF2–SF5), and pure compounds (**1** & **2**) isolated from borututu bark in comparison with selected antioxidant herbs (horse apple (BPE), milk thistle (SME), and green tea (GTE)) and spices (nutmeg (MFE)).

Extraction of borututu bark powder (500 g) yielded 102.7 g of total methanolic extract (TME), of which 57.2 g were used for subsequent fractionation. The utilized weight (57.2 g) subsequently yielded four fractions obtained by successive solvent extraction of TME-coated silica (HF, 0.64 g; CF, 5.63 g; EF 6.58 g; MF, 29.45 g). The ethyl acetate fraction (EF) showed the highest DPPH scavenging activity followed by the methanol fraction (MF). This was qualitatively verified by a visible inspection of DPPH-dipped TLC plates and quantitatively determined by 96-well plate DPPH-scavenging assays as shown in Figure 2A and Figure 3, respectively. Thus, EF was further investigated to identify active compounds. Flash chromatography of an aliquot of EF (1.40 g) resulted in five subfractions (SF1–5) with SF4 (0.17 g) and SF5 (0.14 g) showing the highest activity (Figure 3). TLC analysis of SF4 and SF5 showed two major compounds that bleached the purple background after dipping in DPPH solution (Figure 2B). Each subfraction was subjected to centrifugal preparative TLC (Chromatotron), resulting in one major compound per subfraction (SF4: Compound **2**, 0.07 g; SF5: Compound **1**, 0.03 g). Although the CHCl_3_ fraction (CF) exhibited lower DPPH scavenging activity, its TLC profile showed two orange spots and one faint yellow spot that were isolated by preparative flash chromatography (Isolera One^®^) of an aliquot (0.48 g) to yield Compounds **3** (0.03 g), **4** (0.03 g), and **5** (0.005 g).

### 3.2. Identification of Pure Compounds

**Figure 4:**
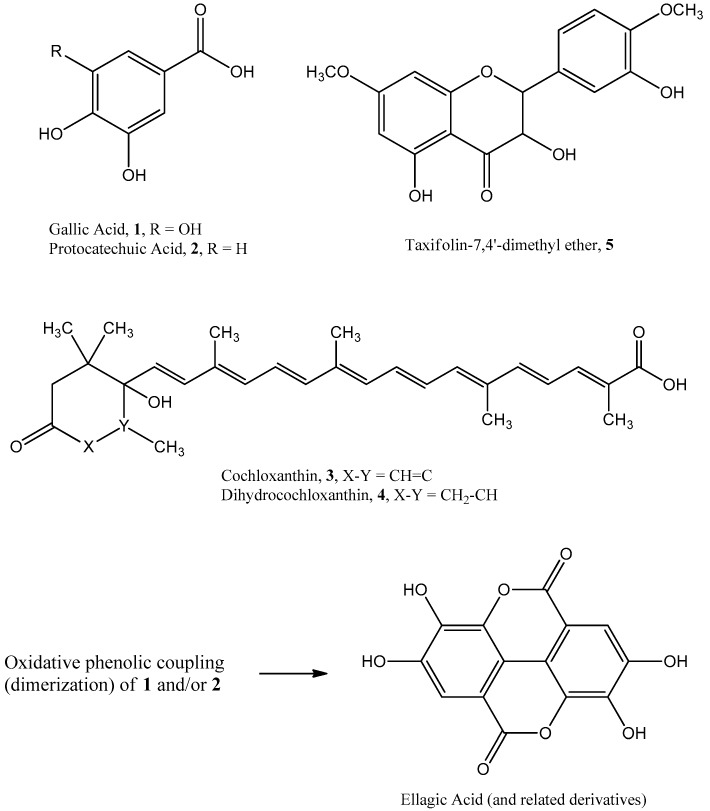
Chemical structures of phenolic compounds and carotenoids isolated from *C. angolensis* and biosynthetic relationship of **1** & **2** to ellagic acid.

Spectroscopic data from ^1^H and ^13^C NMR and MS analysis of **1** and **2** matched reported literature for 3,4,5-trihydroxybenzoic acid (gallic acid, M.W. 170) [20] and 3,4-dihydroxybenzoic acid (protocatechuic acid, M.W. 154), respectively [21]. Additionally, spectroscopic data for **3** and **4** matched those reported for the apocarotenoids cochloxanthin (M.W. 462) and dihydrocochloxanthin (M.W. 464), respectively, previously reported in *C. tinctorium* [15]. Spectroscopic data for **5** also matched literature values for taxifolin-7,4′-dimethyl ether (M.W. 332) [22]. Thus, to the best of our knowledge, Compounds **1**–**5** are hereby reported for the first time in *C. angolensis* (Figure 4).

### 3.3. Evaluation of Antioxidant Activity

Compounds **1** and **2** had the highest free-radical scavenging activity of all tested samples (Figure 3). It is to be noted that antioxidant ellagic acid (Figure 4) derivatives reported earlier by Ferreres et al. are dimeric analogs of **1** and **2** and are probably responsible for the observed free-radical scavenging activity of MF [5]. When compared to the antioxidant herbs—green tea (GTE), milk thistle (SME), and horse apple (BPE)—the DPPH scavenging activity, expressed as percent Trolox equivalence, of the total borututu extract was approximately 10%, 50%, and 100% that of GTE, SME, and BPE extracts, respectively (Figure 3). The DPPH scavenging activity of borututu was equivalent to that of nutmeg (MFE), which is also reported to possess antioxidant activity (Figure 3) [23]. As shown in Figure 3, the DPPH activity of borututu samples gradually increased with each level of fractionation, reaching the highest activity with pure compounds. Thus, by adopting free-radical scavenging-guided fractionation, the most active antioxidants of borututu bark were isolated and identified as gallic and protocatechuic acid (**1** and **2**, respectively).

### 3.4. HPLC Analysis of Extract, Fractions, and Pure Compounds

**Figure 5:**
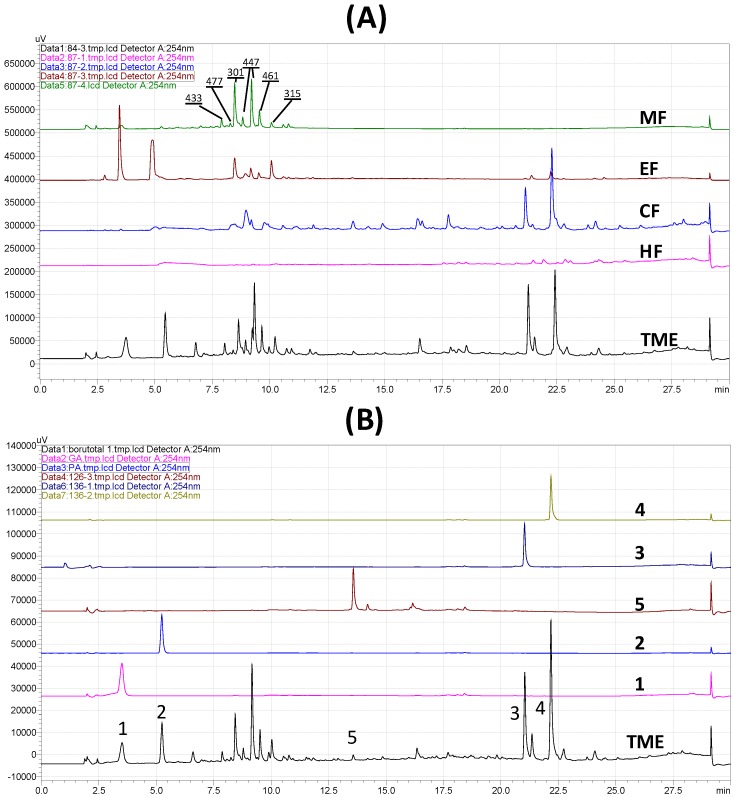
Qualitative HPLC analysis of borututu bark. (**A**) Fingerprint of total methanolic extract (TME) and successive solvent fractions: chloroform (CF), ethyl acetate (EF), and methanol (MF; pseudomolecular ions [M−H]^−^ of ellagic acids marked on peaks). (**B**) Pure compounds isolated from CF and EF: gallic acid (**1**), protocatechuic acid (**2**), cochloxanthin (**3**), dihydrocochloxanthin (**4**), and 7,4′-dimethyltaxifolin (**5**).

HPLC fingerprinting was initially performed on borututu TME in order to identify different phytochemical markers that may be used to characterize the extract and to guide the subsequent isolation of these markers. Each solvent fraction of the total extract (CF, EF, and MF) contained one or more of the markers identified in TME. Figure 5A shows the fingerprint of the total extract and the chromatographic profiles of active subfractions with major markers identified in each. Fraction CF contained **3**, **4**, and **5** (retention times: 21.1, 22.3, and 13.6 min, respectively). Fraction EF showed **1** and **2** as its major constituents (retention times: 3.5 and 5.3 min, respectively). Fraction MF showed a cluster of peaks eluting between 7.5 and 11.5 min that were not isolated as pure compounds. However, these peaks showed the pseudomolecular ions [M−H]^−^ 301, 315, 433, 447, 461, and 477 corresponding to ellagic acid and its glycosides and/or methyl analogs reported by Ferreres and co-workers [5]. The hexane fraction, on the other hand, did not possess any antioxidant activity and did not contain any significant UV-active markers. The same HPLC method was subsequently utilized to further confirm the purity of isolated compounds and to demonstrate their potential use as quality markers for borututu products (Figure 5B).

## 4. Conclusions

Borututu bark possesses significant in vitro free-radical scavenging activity that supports its use as an antioxidant herbal dietary supplement. The main active constituents were identified as gallic acid (**1**) and protocatechuic acid (**2**). Additionally, the two apocarotenoids cochloxanthin (**3**) and dihydrocochloxanthin (**4**) and the flavanoid taxifolin-7,4′-dimethyl ether (**5**) were identified as significant constituents of the bark. All identified compounds are reported for the first time in *C. angolensis*. Compounds **1**–**4** can serve as analytical markers for quality control of borututu products in addition to previously reported ellagic acids [5]. Development of quantitative analytical methods and further investigation of the pharmacokinetics/dynamics of borututu bark are thus warranted.
